# Bio-economic potential of ethno-entomophagy and its therapeutics in India

**DOI:** 10.1038/s41538-024-00260-3

**Published:** 2024-03-09

**Authors:** Wahengbam Deepanita Devi, Rajkumari Bonysana, Kabrambam Dasanta Singh, Arunkumar Singh Koijam, Pulok Kumar Mukherjee, Yallappa Rajashekar

**Affiliations:** Insect Bioresources Laboratory, Animal Resources Programme, Institute Bioresources and Sustainable Development (IBSD), Takyelpat, Imphal West, 795001 Manipur India

**Keywords:** Animal physiology, Urban ecology

## Abstract

Insects are the largest group of arthropods with the highest faunal diversity of over a million species. Apart from many other roles in the environment, the aspect of several insects being used for human consumption (entomophagy) and as traditional medicine (entomotherapy) by different communities of the world holds special significance for countering global food crisis. The enormous insect resources contribute a reasonable share in improving the livelihoods of many entomophagy practicing communities. Considering this significance, the present review emphasizes the bio-economic potential of insect resources. An overview of entomophagy practices in India; benefits towards the environment, humans and animals; insect species used in entomophagy along with therapeutic importance, nutritional, physical, chemical, and microbiological hazards; farming and mass production technologies; legal status and socio-economic implications in Indian scenario have been presented. Traditionally tested and accepted therapeutic use of edible insects have been reported to cure various disease conditions and calls for scientific exploration and validation to rediscover promising medical products of modern medicine. Edible insects as an alternative food need to be popularized in India with a new policy or regulation to harvest and sell insect-derived food products with proper safe consumption demonstrations. Considering the facts that insects reproduce in large numbers at a faster rate, require less land, water and other resources for farming, and economically and ecologically sustainable harvesting can be done in a short time, insect farming can offer revenue and rural job opportunities in developing countries, especially in India. Therefore, the traditional use of insects as food and medicine has tremendous potential to enhance the economy and living standards.

## Introduction

Insects provide various advantages to the ecosystem and human well-being^1,2^. Out of the million recorded insect species, over 2141 species are edible and are traditionally consumed as food by some ethnic communities, especially in Africa, Asia, Australia, and Latin America^3,4^. Insects are widely consumed by over 2 billion people worldwide in 113 nations due to their unique flavor and several health benefits derived from their diverse nutrient content. A list of some common edible insects is depicted on Table 1^5–7^. They are also utilized as traditional medicine^8,9^. The use of insects and their products for therapeutic purposes is known as entomo-therapy^10^. Several records exist regarding entomophagy in diverse ethnic communities and geographical areas. Insect and habitat diversity, their abundance, and the living standards of communities account for different entomophagy practices in different countries^6^. These traditionally based edible insects are believed to be healthy foods and are a storehouse of various nutrients because of their incredible source of fats, energy, proteins, vitamins, minerals, and fiber which are essential for human beings^11^. Thus, edible insects have a variety of health benefits that make them a popular food among nutritionists, health workers, and physicians. However, there are several factors that determine the safe practice of entomophagy which includes allergenicity, mycotoxin, pesticides and microbial contamination, heavy metals, etc.^12^. These factors need to be addressed properly to ensure safe consumption of edible insects. Though the edible insect market has huge potential to improve global food security and the economy, it still lacks an explicit regulation to control production, processing, and marketing of edible insects. In spite of such lacunae, insect resources can act as a potential bio-resource for expanding the bio-economy of a nation. Bio-economy literally means earning from natural resources in a sustainable way. Relevant articles, books and other sources; retrieved from DeLCON DBT-E Library Consortium and Google Scholar, using the key words such as ‘edible insects’, ‘entomophagy practices in India’, ‘insect farming’ ‘edible insect regulation’, ‘nutrition from insects’, etc. were used in the compilation of this review article. Out of several thousands only 85 related articles were selected for the present paper.

**Table 1:** List of edible insects

Sl. No	Order: Family	Scientific name	Reference
1.	Coleoptera: Cerambycidae	*Batocera rubus*	^5^
2.	Coleoptera: Cerambycidae	*Batocera parryi*	^5^
3.	Coleoptera: Cerambycidae	*Orthosoma brunneum*	^5^
4.	Coleoptera: Dytiscidae	*Cybister limbatus*	^5^
5.	Coleoptera: Dytiscidae	*Cybister tripunctatus lateralis*	^5^
6.	Hemiptera :Aetalionidae	*Darthula hardwickii*	^5,6^
7.	Hemiptera: Belostomatidae	*Lethocerus indicus*	^5^
8.	Hemiptera: Dinidoridae	*Aspongopus nepalensis*	^5^
9.	Hemiptera: Dinidoridae	*Coridius chinensis*	^5^
10.	Hemiptera: Pentatomedia	*Tessaratoma javanica*	^5^
11.	Hymenoptera: Apidae	*Apis dorsata*	^6,7^
12.	Hymenoptera: Apidae	*Apis florea*	^5^
13.	Hymenoptera: Formicidae	*Oecophylla smaragdina*	^5,7^
14.	Hymenoptera: Vespidae	*Vespa bicolor*	^6^
15.	Hymenoptera: Vespidae	*Vespa mandarinia*	^5,6^
16.	Lepidoptera: Bombicidae	*Bombyx mori*	^7^
17.	Lepidoptera: Crambidae	*Omphisa fuscidentalis*	^5^
18.	Lepidoptera: Hesperiidae	*Erionota torus*	^5^
19.	Lepidoptera: Saturniidae	*Antheraea assamensis*	^5^
20.	Mantodea: Mantidae	*Hierodula coarctata*	^5^
21.	Mantodea: Mantidae	*Tenodera sinensis*	^5^
22.	Odonata: Aeshnidae	*Crocothemis servilia servilia*	^5^
23.	Odonata: Libellulidae	*Neurothemis fulvia*	^5^
24.	Odonata: Libellulidae	*Orthetrum Sabina sabina*	^5^
25.	Odonata: Libellulidae	*Pantala flavescens*	^5^
26.	Odonata: Libellulidae	*Potamarcha congener*	^5^
27.	Orthoptera: Acrididae	*Oxya hyla*	^5^
28.	Orthoptera: Gryllidae	*Acheta domesticus*	^5^
29.	Orthoptera: Gryllidae	*Tarbinskiellus portentosus*	^5^
30.	Orthoptera: Gryllotalpidae	*Gryllotalpa orientalis*	^5^
31.	Orthoptera: Tettigonidae	*Elimaea securigera*	^5^
32.	Orthoptera: Tettigonidae	*Pseudophyllus titan*	^5^

## Entomophagy in India

**Fig. 1:**
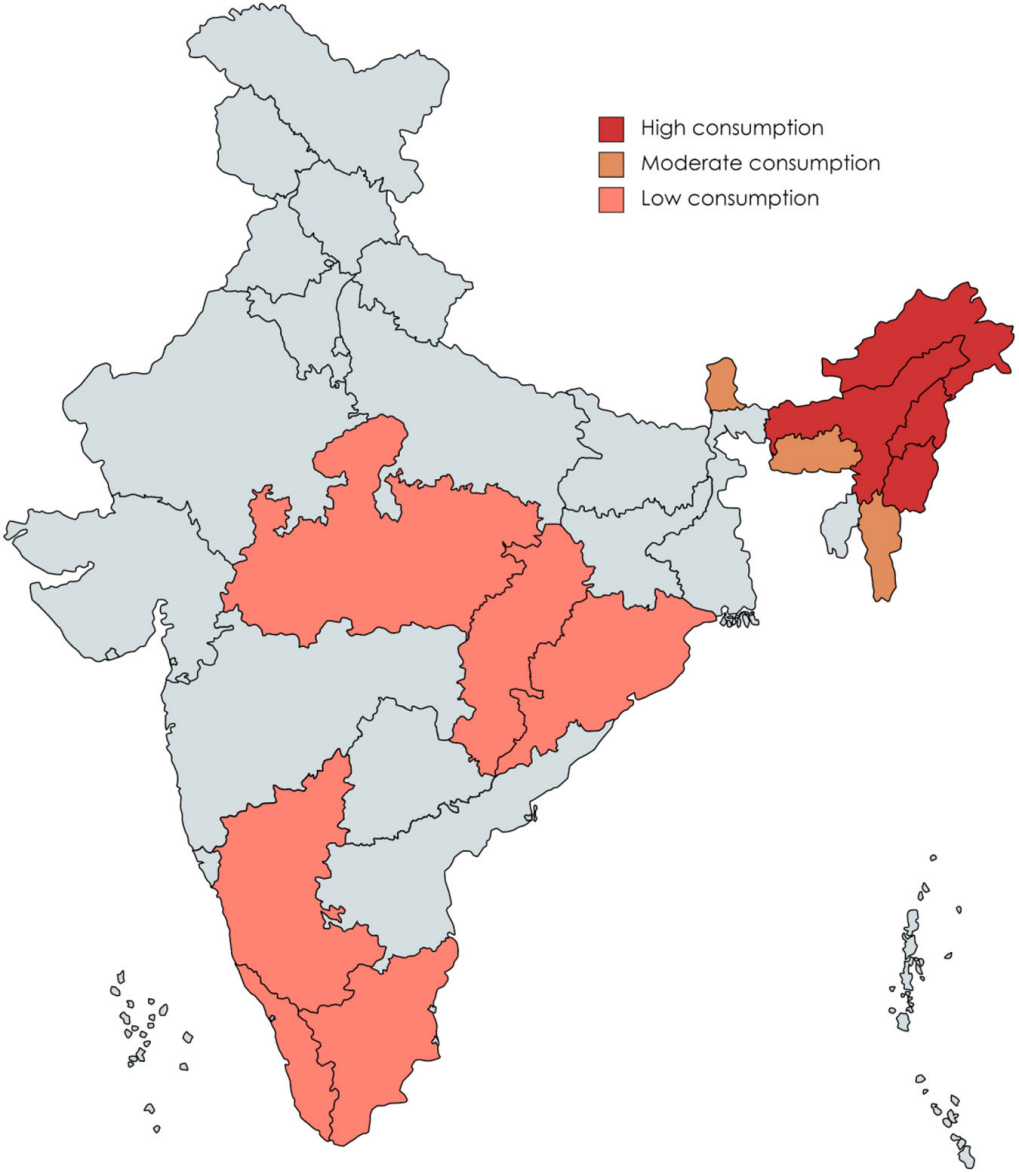
Map of India. Map showing major Indian states consuming edible insects.

**Fig. 2:**
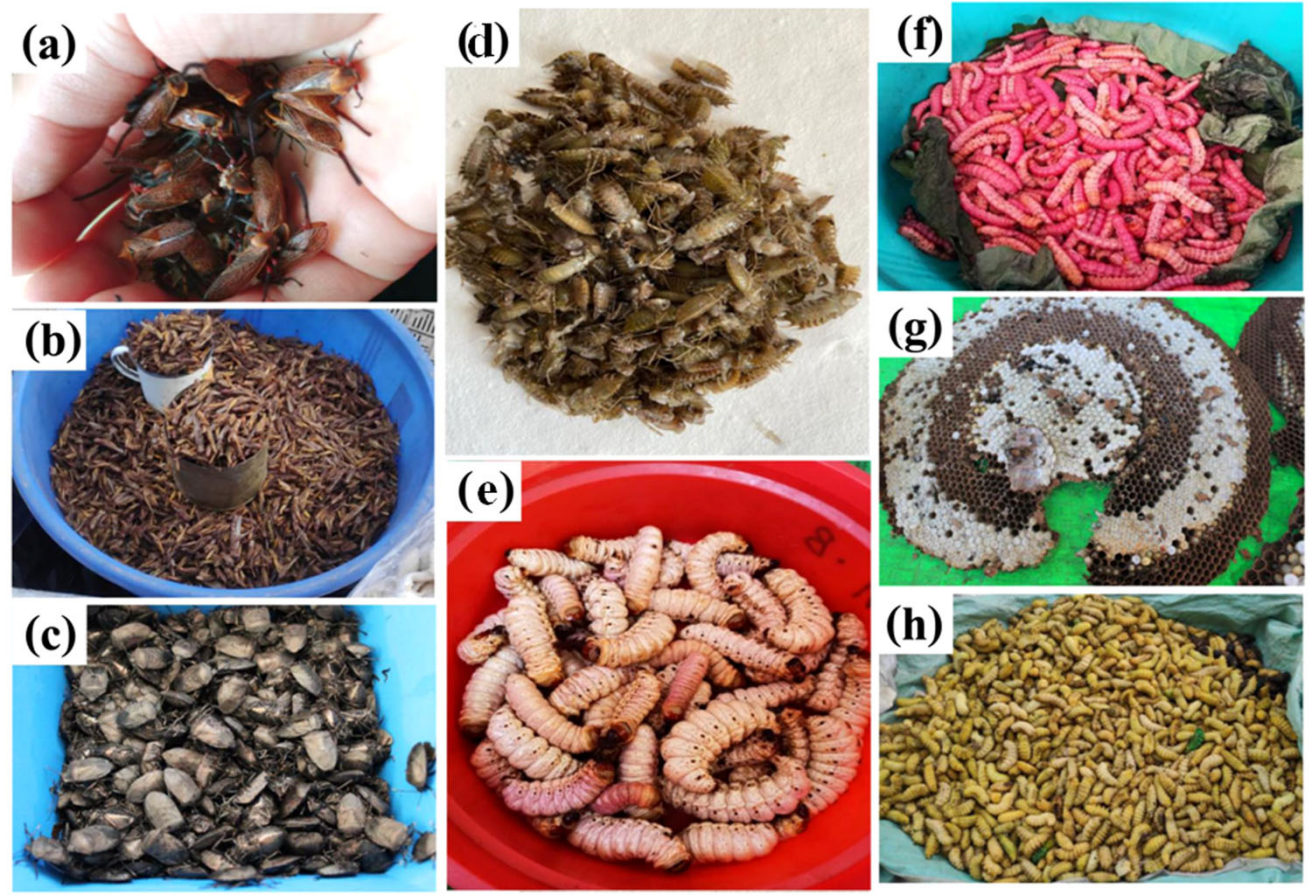
Representative edible insects collected from wild and farm. **a** Treehopper, (**b**) Grasshopper, (**c**) Stink bug, (**d**) Nymph of dragonfly, (**e**, **f**) Wood borer, (**g**) Wasp, (**h**) Silk worm.

**Fig. 3:**
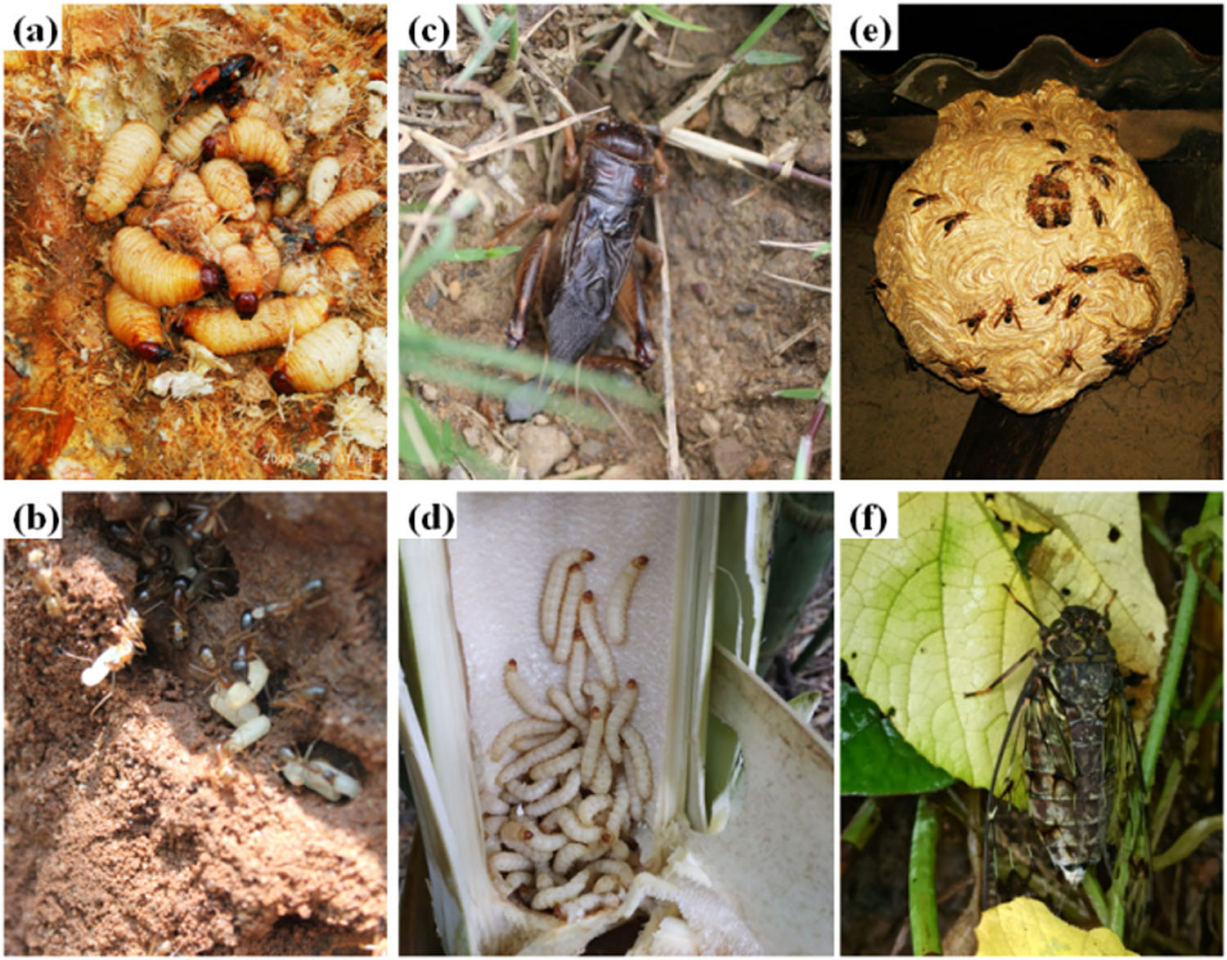
Edible insects at their natural habitats. **a** Palm weevil, (**b**) Ant, (**c**) Cricket, (**d**) Bamboo borer, (**e**) Wasp, (**f**) Cicada.

Consumption of insects is not a new concept but an ancient practice inherited from the ancestors and still practiced in this new world. This inherent knowledge has to be preserved by recording it. However, evolving food habits and human intervention on insect habitats has resulted in declining insect population. In India, entomophagy is practiced by different ethnic communities in Assam, Arunachal Pradesh, Chhattisgarh, Karnataka, Kerala, Manipur, Madhya Pradesh, Meghalaya, Nagaland, Orissa, Sikkim, Tamil Nadu, and Tripura^13^ (Fig. 1). Among these, the ethnic community of Assam, Arunachal Pradesh, Nagaland and Manipur consume the highest. More than 300 insect species are consumed by different communities in India with Coleoptera being the most preferred order, followed by Orthoptera and Hemiptera^14^. The most common edible insects in India are bees, wasps, termites, wood borer larvae, grasshoppers, silk worm larvae, palm weevils, etc. (Fig. 2). Nowadays, consumption of edible insects is decreasing in some areas due to changes in lifestyle and the less availability of insects. Most of edible insects are available in wild and present in different ecological niche (Fig. 3). Entomophagy practices in different regions of India are as follows:

## Northeast India

Northeast India comprises a diverse ethnic group of more than 220 communities with unique cultures and is known for its biodiversity hot spot^15^. Along with a diverse community and culture, the North East Indians have distinct food habits from other regions of India. The tradition of entomophagy is reported from all the regions of Northeast India. The ethnic people of Arunachal Pradesh, Assam, Manipur, and Nagaland consume more varieties of edible insects than the people of Tripura, Meghalaya, and Mizoram. Chakravorty^13^ reported a total of 294 edible insects, i.e., 158 from Arunachal Pradesh, 41 each from Manipur and Nagaland, 38 from Assam, 16 from Meghalaya. Similarly, Thangjam et al.^15^, reviewed around 200 edible insects, of which 92 species are from Nagaland, 69 species from Manipur, 67 species from Assam, 65 species from Arunachal Pradesh, and only some from Meghalaya, Mizoram, and Tripura. So far, only one edible insect i.e, *Udonga Montana*, from Tripura and Mizoram, and three edible insects, namely *Apis* species, *Solenopsis* species and *Tetragonula iridipennis* from Sikkim, have been reported^16^. The studies from Assam and Arunachal Pradesh show that maximum numbers of insects consumed by the locals were from the Coleoptera order^17^. However, in Manipur highest consumption was observed from the order Hymenoptera with 20 insect species^6^. Similarly, Mozhui et al.^5,18^ reveals that in Nagaland, the insect with highest consumption belongs to the order Hymenoptera and Orthoptera.

## East India

In Rayagada district, Odisha, eggs of red ants along with the worms found in date palm are consumed by locals. Additionally, the ethnic communities of Koraput, Kandhamal, Keonjhar, Sundergarh, and Mayurbhanj districts consume roasted red ants and termites as snacks or with rice. They also consume ants which are fried with salt, chillies, spices, and mustard oil^19^. Kai chutney which is prepared from red ants (*Oecopylla smaragdina*) in the Mayurbhanj district of Odisha, has been sought for GI tag^20^.

## Central India

In Chhattisgarh, red ant chutney is popular among the Gond tribals^19^. From Madhya Pradesh, Bhowate and Kumar^7^ recorded 10 insect species namely, *Oecophylla smaragdina, Polistes carolina, Apis dorsata, Apis indica, Sceliphron spp., Bombyx mori, Mylabris pustulata, Microterm esobesi, Hieroglyphus banian* and *Pachliopta aristolochiae* of different families as food of the tribals of Satpura Plateau. Additionally, since 1946 the silk federation has been producing canned silkworm pupae, which are used for preparing pickle and as a topping of pizza^19^.

## South India

The different ethnic groups of Kothagiri, Anamalai Hills, Bodi Hills, Theni District, Kodaikanal, Sathyamangalam Forest, and Gudalur Hills from Tamil Nadu practice entomophagy, where adult Macrotermites species; adults, eggs, and larvae of *Oecophylla smaragdina*; larvae of *Apis spp*. and adults, eggs, and larvae of other *Hymenoptera* species are traditionally consumed by cooking. Larvae of *Apis* spp. are also eaten raw. *Oecophylla smaragdina* and *Hymenoptera* species are used to prepare soup, locally called rasam and thuvaiyal^21^. From Kerala, 4 species of Hymenoptera i.e., *Apis cerana indica, A dorsata, A florae* and *Oecophylla smaragdina*, and 1 from Orthoptera, i.e., *Patanga succinata* were reported as food^22^. Roasted *Odontotermes obesus* is taken as food with maize or rice by Koya and other communities residing in Warangal district of Telangana^23^.

## Benefits of Edible insects

### Environmental benefits

The Agriculture sector, especially livestock production, contributes around 80% of the global greenhouse gas emissions, which has a detrimental impact on human health and threatens food production in many nations^24^. Considering this, insect farming for alternative protein production has been approached as it has less impact on human health and the environment^25^. Moreover, compared to conventional livestock, commercially raised edible insects, like house cricket (*Acheta domesticus*), yellow mealworm (*Tenebrio molitor*) and migratory locust (*Locusta migratoria*) perform better in terms of direct emissions of GHG^26^. When feed conversion, land use, and water use are taken into account, the environmental impacts of insect production are substantially less than those of other animal types, especially chickens, pigs, and cattle^25^. Though insects farming requires less feed, land, and water, the protein content are almost similar with that of livestocks; for example, the protein efficiency of cricket is 154 g/kg and the protein content of beef, pigs, and poultry is 190, 150, and 200 g/kg of edible mass respectively^26^. Insects play an important role in bio-waste degradation. A variety of insects, including the black soldier fly (*Hermetia illucens*) and some species of mealworm can also be utilized for this purpose^26^. They may turn animal waste from dairy, poultry, and swine into body mass, reducing dry matter mass by up to 58%^27^. They have the ability to recycle agricultural and forestry wastes into premium food or animal feed (such as palm weevils) which is another advantage of entomophagy^28^ and enhances the nutrient content of agriculture and forest areas. A variety of ecological functions provided by insects are essential for human survival. As an illustration, insects are crucial to plant reproduction^26^. Most of the fruits and vegetables we consume and the renewal of many feed crops used by cattle, depend on insect pollination. At least 80% of these crops are pollinated by wild bees and other wildlife, whereas only 15% of them are supported by farmed honey bees.

### Nutritional benefits

**Fig. 4:**
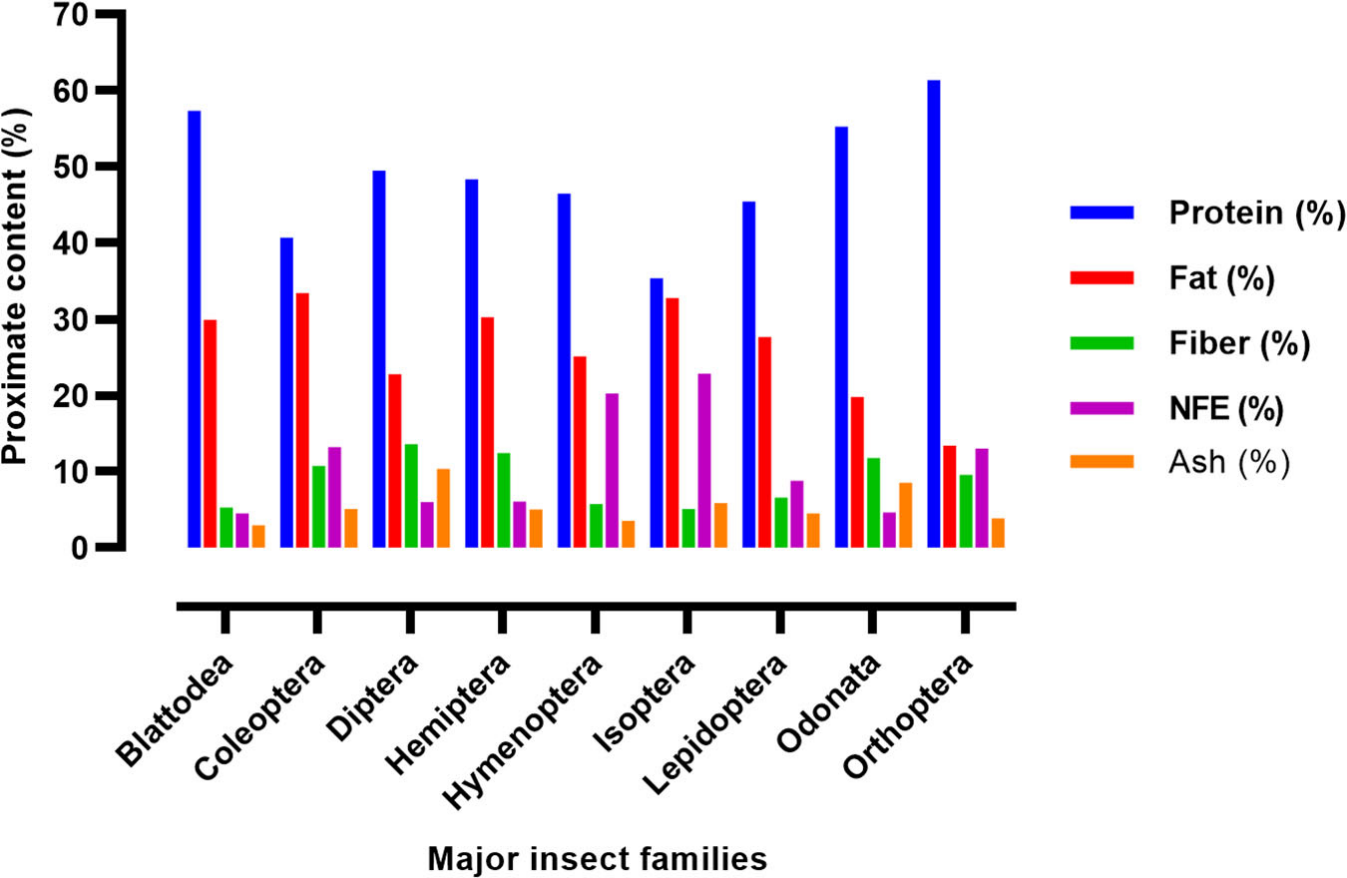
Percentage proximate content major insect families.

In general, edible insects are incredibly rich in nutrients. They are an important source of proteins, which consist of essential amino acids ranging between 7–91% of protein^29^. The protein content of legumes, dairy products, and other meats like chicken, pork, eggs, beef, or lamb is comparable to or less than that of edible insects^30^. According to reports, grasshoppers, crickets, and locusts (Orthoptera) are abundant and alternative sources of protein^31^. Edible insects offer optimum levels of essential amino acids, including methionine, cysteine, lysine, and threonine^32^. The order Blattodea shows the highest concentration of lysine, valine, methionine, arginine, and tyrosine when compared with other studied orders^33^. The amino acid contents of edible insects vary depending on the insect type^34^. Lipids make up the second-largest percentage of edible insects which are crucial to their nutritional worth^31^. The proximate contents in edible insects varies by species (Fig. 4) and even fat content also varies, ranging from 7 to 77 g/100 g dry weight; however, termites, caterpillars, and palm weevil larvae have the highest fat contents^35,36^. Edible insects contain polyunsaturated fatty acids (PUFA) including linoleic and linolenic acids^37^. *Chondacris rosea* and *Brachytrupes orientalis* are common species of Arunachal Pradesh which contained saturated fatty acid (SFA), monounsaturated (MUFA) and Polyunsaturated (PUFA)^38^. The fat contain of edible insects varies by species, metamorphic stages, as the larva and pupa have a high fat content as compared with the adult stage^39^. Besides proteins and fats, edible insects contain various minerals such as iron, zinc, potassium, sodium, calcium, phosphorus, magnesium, manganese, and copper, and vitamins like Vitamin A, B1, B2, B3, B6, B12; Vitamin C and D^26^. For examples in 100 g sample of *Rhynchophorus phoenicis* mineral compositions satisfy the Recommended Daily Allowance values for iron, zinc, copper, manganese, and magnesium^40^. In most of the edible insects, thiamine (vitamin B1) levels range from 0.1 to 4 mg/100 g, riboflavin (vitamin B2) level ranges from 0.11 to 8.9 mg/100 mg whereas in whole-meal bread content only 0.16 and 0.19 mg/100 g of B1 and B2, respectively^35^. Meanwhile, edible insects are a valuable source of calories, contributing between 290 and over 750 kcal/100 g^41^.

### Social impact

Insect consumption is continuously growing as interests are increasing in the new resource. The exports of edible insects are quite infrequent and local^33^. People often follow their traditions; the insect groups eaten can occasionally differ noticeably, even between surrounding places^42^. To influence public perspective and acceptance, the traditions should be upheld^33^. Some species, such as the black soldier fly, house fly, and yellow mealworm, are effective at bio-converting organic waste like rotten fruits during their growth by converting the low-nutritive waste into high-nutritive products^43–45^. Additionally, it is used as fish, poultry, cattle, and pig feed, which is practical, inexpensive, and environmentally friendly^25,26^. It would be beneficial to create networks between farms and industries, which might boost their ability to produce. It makes financial sense for established firms to create new medicines and healthcare items based on insect extracts^33^. Several works have emphasized the benefits of edible insects for health and well-being as well as for industrial purposes^26^. Without changing the taste preference of items that people are familiar with, the appropriate extraction and usage of the extracted insect proteins can create food with the needed qualities^46^.

### Insects farming: approaches and application

**Fig. 5:**
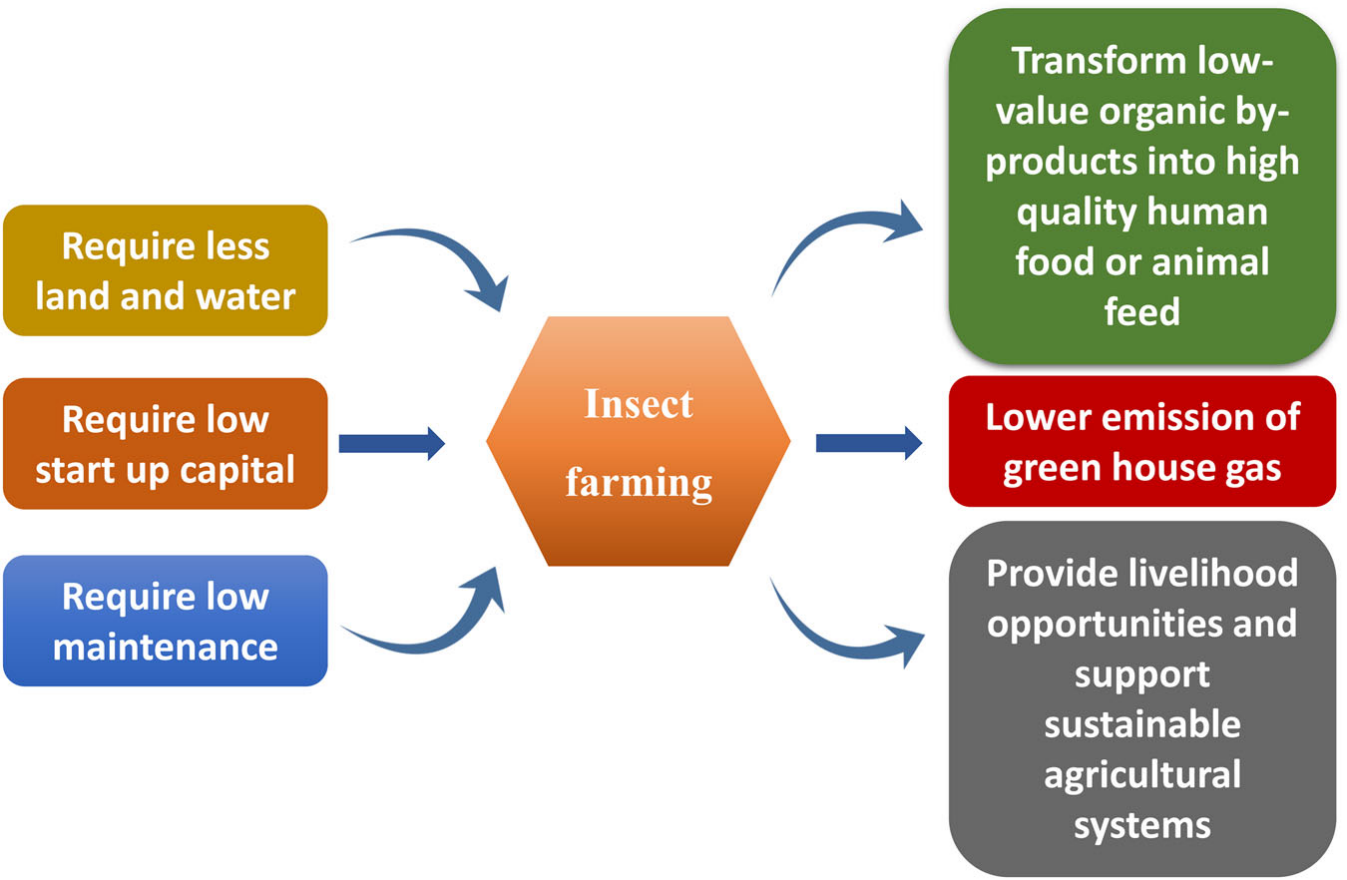
Insect farming; approaches and application.

Insects are usually collected from forest and agricultural areas without maintaining sustainable harvesting. If this continues, the insect population is bound to decrease. So, the idea of insect farming has come up. Another main reason for insect farming is pesticide accumulation in insects like crickets and grasshoppers from agricultural fields. Farming insects is rearing and breeding of insects as livestock and harvesting them for human consumption or as feed for livestock. Hence, sustainable insect farming needs to be implemented. Moreover, insect farming can be done either on small-scale farms or in large-scale facilities and does not require high start-up capital. The other main benefits of insect farming compared to livestock production are that it requires less land and water, lower emissions of greenhouse gases, high rates of feed conversion, and transforming low-value organic to high-quality by-products for human food or animal feed^47^ (Fig. 5). The edible insect-based industries are still underway in India. Starting insects farming on a large scale will lead to the opening of new industries, provide opportunities for employment, and increase the regional income of the country^33^. Cricket farming is practiced in Manipur, India, where thousands of crickets are sheltered in a modified dark environment.

### Animal feed

Edible insects are also used as animal feed. Insects like black-soldier flies larvae, common house fly larvae, crickets, and mealworms are the most common insects used for animal feed production^48^. The high protein content of edible insects makes them one of the potential sources of animal feed. Edible insect dry products contain around 50 to 80% protein which is essential for animal (livestock, fish, and pet) nutrition. The animal feed products from meat meal, fishmeal, and soybean meal are more expensive than insect-based feeds. Because of this, insect-based proteins are in higher demand and are popular around the world^49^.

In India animal feed industry mainly uses maize, corn and soya for feeds production. The rising prices of these crops result in increased prices of animal feed eventually reducing the profit margins of feed producers. This creates hurdles for many small and medium sized feed producers to sustain their profit. Hence, many companies look for new alternative raw materials and edible insects present one of the best options to decrease production cost^50^.

### Waste management by insects

The process of managing food waste by insects is known as bioconversion. Insects have the ability to break down and convert organic material including agricultural waste such as fruits and vegetable products during their mass production under controlled conditions. They consume the waste and convert it into insect biomass along with other valuable by-products like pharmaceuticals, biofuels, lubricants, and fertilizer^48,51^. This bioconversion has a high potential to fulfill the future protein demands, as many organizations including FAO, European Union, and U.S. Department of Agriculture also support the use of insect protein as a good source for feeding future populations^26,52,53^. Further, the frass produced from insect farming can be used as fertilizers, which reduces pathogenic microbes and pesticide use^54^.

### Ethno-entomotherapy practice in India

Entomo-therapy practices in India were reported from some indigenous communities of Assam, Arunachal Pradesh, Chhattisgarh, Kerala, Manipur, Madhya Pradesh, Nagaland, and Tamil Nadu. Such practices are also mentioned in Materia medica of Ayurveda, where about 15–20% of medicines are of animal products including insects^8^. Among the Indian ethnic communities, insects have been used in various ways. Some edible insects are also used as sources of therapeutic material. Many authors reported the therapeutic benefit of insects from different regions^14^. Senthilkumar et al.^55^ and Lokeshwari and Shantibala^56^, reviewed a total of 76 and 9 medicinal insect species respectively, found in India that are utilized by the local population to treat a variety of illnesses. Some of the insects they listed are edible. Kemprai et al.^57^ recorded entomo-therapeutic practices of seven different ethnic groups of Semkhor, Assam. Forty-one species of medicinal insects were recorded for the treatments of at least 53 human ailments, where the most frequently mentioned were coughs, gastritis, rheumatoid arthritis, stomach ache and wound healing. From the recorded 41 medicinal insects, 38 species were highly accepted as food. *Vespa affinis* L. is one of the common edible insects of the North-East region of India. The antioxidant potential of the aqueous extract of this edible insect may have therapeutic activities in oxidative stress-associated health disorders^58,59^. Silkworm pupae are also one of the most consumed edible insects mostly in Assam, Arunachal Pradesh and Nagaland. The peptides derived from pupae protein hydrolysates have great potential nutritional approaches against hypertension and related cardiovascular diseases^58^. The honey of a stingless bee, *Lepidotrigona arcifera* (Cockerell) is reported to have ethnomedicine property by the Nepali community of Darjeeling foothills of West Bengal. The use of *Lepidotrigona* honey either alone or in combination with other ingredients (some plant parts, cow urine, milk, etc.) has been found to cure about 18 different ailments (ranging from simple cold and cough to diseases like cancer) traditionally^60^. From South India, Dixit et al.^61^ recorded 42 animal species, 12 of which were of insects that are used in traditional medicine. Few entomophagy insects from the Western Ghats’ Attapadi Hills, such as *Apis cerana indica*, *A. dorsata*, *A. florea*, *Oecophylla samragidna*, *Periplanata orientalis*, *Bombyx mori*, and *Vespa orientalis* were among the vertebrates which are known to have medicinal potential^62^. In Kerala, more than 15 different diseases, including anemia, asthma, rheumatoid arthritis, malaria, ulcer, and more, have been treated using insects and insect resources like honey, termites, ants, wasps, mole crickets, and black beetles^63^. From Tamil Nadu, Ranjitsingh and Padmalatha^64^ and Samuel et al.^21^ documented 11 species of therapeutic insects used by traditional healers in the Tirunelveli region and 2 entomotherapy species in the Kothagiri area of the Nilgiris district respectively. Likewise, Bagde and Jain^65^ reported *Periplanata americana* for the treatment of asthma by the community of Chhindwara district. Similarly, Bhowate and Kumar^7^ recorded 11 numbers of therapeutic insects used by the people of Chhindwara and Betul districts of Madhya Pradesh. Their studies reported that insects like *Trombidium grandisimum* can cure pneumonia by mixing one of the two parts of the dry insects with one teaspoonful of milk to children, one dry insect is administered orally for fever; live ants (*Oecophylla smaragdina*) crushed with salt, red chillies and mustard oil are eaten with rice to prevent gastritis; fume of a wax hive of *Polistes carolina* applied on piles and general wound; Ash of *Bombyx mori* larva mixed with honey is applied on the chest for curing pneumonia. Compared to other states, Oudhia^66^ recorded more than 500 insects and mite species which have been used as traditional medicine practiced by over 3500 traditional healers of Chhattisgarh. Additionally, boiled nymph of *Crocothemis servilia servilia* is eaten for the treatment of headache and vision problems; the whole body of *Tarbinskiellus portentosus* is roasted and consumed to cure indigestion; soup of adult *Lethocerus indicus* is taken orally twice a day to prevent dry cough; boiled adult *Aspongopus nepalensis* is consumed thrice a day for a week to cure jaundice; cooked adult *Coridius singalanus* is eaten daily for twice a week for the treatment of malaria, viral fever; larvae soup of *Batocera parry* is orally taken to cure aphrodisiac, malaria and typhoid^57^. The entomo-therapeutic practices by different communities of India are different or sometimes similar. Insects are considered as a healthy food having various nutrients, so exploring the medicinally important entomophagy species used for different therapeutic purposes which is traditionally practiced by the people of India is necessary for boosting the economy.

### Bio-economic importance of Edible insects

Bio-economy covers all the sectors and systems related to biological resources by interlinking all the primary production sectors based with all the economic and industrial sectors^67^. Among all the diverse faunal biological resources, insects are the most diverse and abundant. They provide various vital services in an ecosystem^68^. They are a source of foods that contain protein, fat, carbohydrates, vitamins and other essential nutrients. Insects also have therapeutics or medicinal properties for the treatment of minor to major health problems. Insects, therefore, can act as an economic potential by providing raw materials in the industrial sector. Presently, attention has been drawn to this valuable traditional food and medicinal resources, which if explored, are expected to be a sustainable solution for malnutrition. However, insects are used as food and medicine because of the low cost. For many wild species, harvesting is almost free but harvesting takes time as they need to search in large areas. Insect farming requires less space with a high reproduction rate and feed conversion efficiency. In insect farming; they typically eat a variety of low-cost feeds that efficiently transfer energy. Notably, compared to other animals, edible insects have an efficiency of conversion of ingested food (ECI) of between 53 and 73%^69^. The main advantage of insects is that their life cycles are distinctly shorter than other protein sources with less breeding space, low cost investment, time saving, high returns, and can globally contribute to great income opportunities.

### Economic implication

Insect farming can provide rural employment and income generation, either at the small household level or in larger scale industrial operations. In India, where edible insects are in demand and markets are relatively accessible, insect rearing and processing should be encouraged. Selling them as street foods can be encouraged as well for small-scale enterprises.

Edible insects marketing are extending globally with more than 100 companies^34^. India contributes around 8% of the Asia–Pacific edible insect protein market which is increasing rapidly due to the awareness of protein health benefits among the consumers. The highest demand for edible insects product in India is for animal feed followed by food and fertilizer^34,50^. Further, edible insects are consumed for their nutritional properties and therapeutic purposes; therefore, knowledge of nutraceutical potential and therapeutic practice of entomophagy among clients can improve its market potential by producing value added food and pharmaceutical products. For human consumption insects are marketed in local vendor. Depending upon the species, the rate of the insect is also varied. Some insects are harvested for self consumption only. For example, in Manipur insects like tree hopper, cicada, grasshopper, wasp, ants, termites etc. are collected for self consumption, and insects like wood borer larvae, nymph of dragonflies, water beetles, bees, wasps, stink bugs, weevils etc. are sold in market. However, in Nagaland almost all of the edible insects are sold in market and generally in other states the most common marketed insects are silk worm larvae and stink bugs.

### Production concern/production practice

The expenditure and negative impact of insect farming are least as it produces lower greenhouse gases, feeds on bio-waste, and required noticeably less water than that of live stocks. These are the major positive reason that contributes towards insect farming with a huge global potential for animal feed and food production which, will lead to the overall market growth of edible insects. In addition, insect farming also offers employment and revenue to rural people as minimal technical and capital investment is necessary for rearing and harvesting.

### Food safety & food security aspects of edible insects

Insects are known for its high protein contents and other nutritive values. However, the consumption of insects also poses a few risks related to human health. One of the major problems is the pesticide contamination to edible insects collected from agricultural field. Indiscriminate consumption of chemical pesticides on agricultural fields to protect the crops from insect infestation would lead to the accumulation of hazardous chemicals on insects such as grasshoppers, crickets, giant water bugs, water beetles, etc. Consumption of such contaminated insects is dangerous for human health^70^. A more serious problem is the collection of dead insects from the field and selling them in the market that could cause major health issues to unaware consumers. Such cases have been happening in many parts of the world, as supported by a case that happened in Thailand where a group of students were hospitalized after consuming fried insects from a local vendor^71^. Insects may possess certain chemicals that could hamper human metabolic processes. For example, in Nigeria, more than a half decade consumption of African silkworm *Anaphe* spp., especially the pupae cause thiamine deficiency due to presence of a heat-resistant thiaminase^72^. Insects also produce several noxious substances that could keep them safe from predators. Prolonged consumption of such insects will cause accumulation of the noxious substance on the human body, which ultimately compromises the human health^70^. However, most of these noxious substances lose their property when exposed to heat while cooking.

Food allergy to certain individuals is another major cause of concern. Although food allergenicity can be from any food source, edible insects, known for its rich protein content, could easily induce allergic reactions to susceptible people^73^. Especially insects belonging to crustacean such as lobster, shrimp, crayfish, etc. are known to induce allergic reaction to certain individual^74^. Invertebrates’ tropomyosin is a heat-stable IgE-binding allergen in shrimp and cross reactivity study revealed its allergenicity among the other crustaceans, cockroaches, and house dust mites are considered major allergen in crustacean insects^70^. Apart from tropomyosin, other insect allergen includes arginine kinase, α-amylase, glyceraldehyde 3-phosphate dehydrogenase etc.^75^.

Microbial contamination, mycotoxins, heavy metals etc. are other risk factors of consuming edible insects. Microbial contamination of edible insects is understudied and thus limited literature data are available. However, recent efforts have been made to bring light on the occurrence of pathogen transfer to humans and other animals through the consumption of edible insects. Biological contaminants of edible insects include *Baccilus cereus*, *Staphylococcus aureus*, *Escherichia coli*, and *pathogenic Clostridium* spp., *Micrococcus* spp., *Lactobacillus* spp.^76,77^. In a similar way, information regarding the bioaccumulation of heavy metals on edible insects is limited. This problem cannot be underestimated because, if provided substantial time, it could cause serious health problems. There was a report of lead accumulation on grasshoppers from Mexico and was associated with high blood lead levels in Californian children and pregnant women. This was possible because of the insects’ migratory habits which enabled the dissemination of heavy metals^70,76^.

Due to the rise in population and decline in crop production and food availability, food security is a challenge in many emerging and less developed nations^78^. Ironically, the natural resources that have helped achieve the Sustainable Development Goals up to 2030 which priorities achieving food security, ending poverty, and achieving gender equality are being depleted^79^. Insects used as feed for cattle and as food for humans could help ensure food security. The possibility of using insects as food and feed to ensure food security has been evaluated by the Food and Agricultural Organization of the United Nations since 2010. The safety of eating insects has been cited as one benefit. They are frequently picked in places like woodland without the use of pesticide^80^. Many experts are reconsidering diets and various methods for food production, especially on protein production, unpredictability in food prices, rising anxiety over food insecurity, growing concerns about climate change and the significant contributions of the agriculture sector to greenhouse gas emissions, and anxiety over these factors. These reasons create the possibility for entomophagy to provide food security and prospects for the commercial farming or rearing, of insects for food^81^. Edible insects unquestionably contributed significantly to lowering food shortages and nutrient deficiencies throughout the world, particularly in Asia, Africa, and Latin America^8^. Entomophagy is more common in the north-eastern regions of India than its central, northern, and southern regions of India. In North-east India, termites, honey bees, grasshoppers, stink bugs, aquatic insects, and silkworms are frequently consumed because of their high quantities of protein, fat, minerals, and vitamins by indigenous ethnic groups^14,18,82^. This supports the potential of edible insects as a source of a sufficiently nutrient-rich diet in the future, which is highly desirable in the context of food security. Zero hunger, good health and wellbeing, ethical consumption, and responsible production are all made possible by the nutritional profile of insects in combination with more effective use of natural resources and a smaller environmental footprint^83^.

### Indian perspectives: current status of regulation in India

Proper regulation is of prime importance when considering the use of any natural resources. Despite having huge potential with respect to food security and nutritional aspects, regulation of edible insects in both developing and developed countries is not satisfactory. A concrete, well rounded regulatory framework could help in safeguarding the health of the consumers as well as protect the resources from indiscriminate exploitation. Though a few countries like USA, Korea, Thailand, and European countries have their own regulations; their level of regulations is not precise and adequate to ensure the safety to consumers as well as environment. The concept of entomophagy is an age-old practice in many parts of the world, its global acceptance and adoption are still at early stage. Many countries have classified edible insects as novel food, hence regulated under novel food regulations.

It is unfortunate that India does not have any regulatory status related to edible insects despite contributing 8% in Asia Pacific market^84^. As of now, Codex Alimentarius is generally considered for trading farmed insects in the international markets. One of the major limitations in framing a regulatory framework is the lack of complete census data on the consumption of edible insects in the country^83^. The Food Safety and Standards Authority of India (FSSAI) established under the Ministry of Health and Family Welfare should take the initiative to include insect’s products in the list of food items intended for marketing. This will enhance the trade in various levels as well as check for quality of the food.

### Major challenges and future perspective of entomophagy in India

**Fig. 6:**
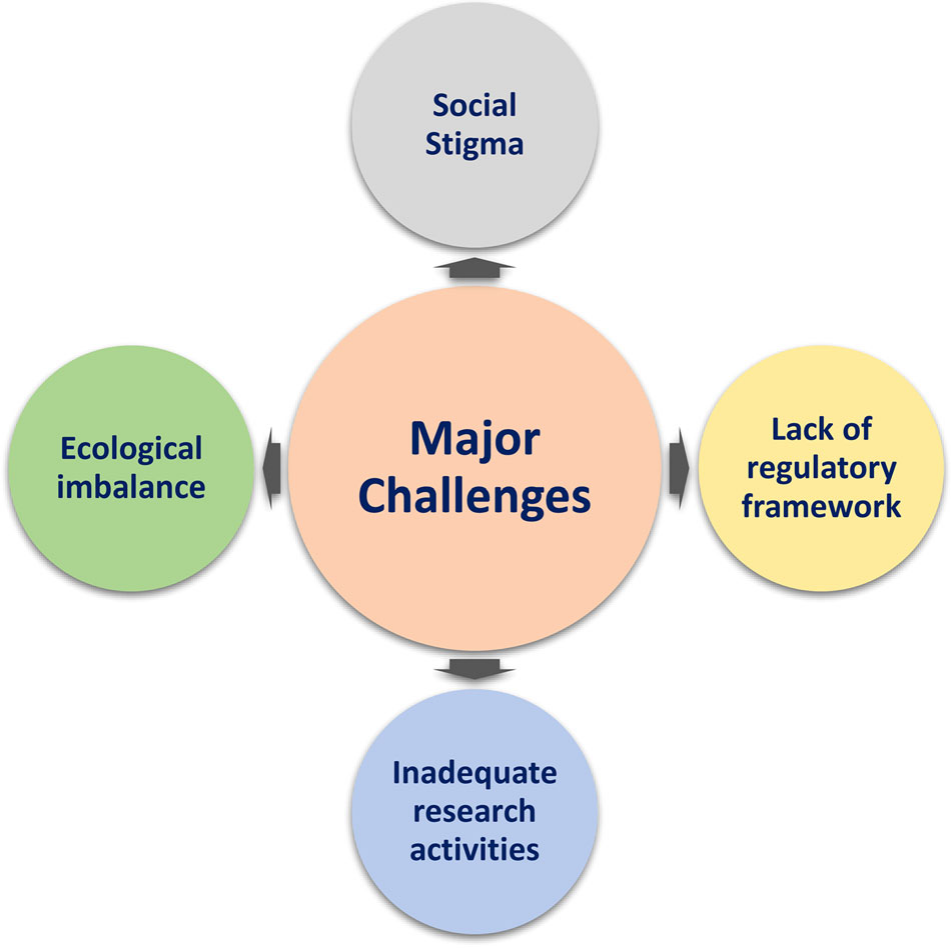
The major challenges of entomophagy in India.

In a country like India where communities with diverse religions and customs coexist together, the practice of entomophagy is not widely accepted (Fig. 6). The social stigma among the people has always restricted the popularity of edible insects’ consumption. Generally, people belonging to schedule tribe and caste prefer the idea of entomophagy because it has been traditionally followed since time immemorial. This is obvious because in the past, people depended on surrounding environment for their food. There is a tendency of linking the practice of entomophagy with people living under the poverty line, which is irrelevant and completely opposite because the cost of those edible insects is high as compared to other normal food. These somehow influence the minds of those who consume insects as an important part of their food habit to openly practice the culture. Proper awareness among the people regarding the cultural importance and nutritional value of edible insects is a prerequisite to make people understand the value we are missing from long time.

Another problem of popularizing entomophagy is its limited availability in nature. Many of the edible insects are wild and are mostly found in forest areas. No medium to large-scale insect farms have been established in India. Therefore, the only source of the edible insects is from their natural habitat. Harvesting insects from forests is also not freely accessible because of several legislations. There is also concern about the imbalance that can happen to the forest ecosystem due to the overexploitation of forest insects. Tree borer is one of the most eaten insects among all those edible insects. They are mainly found inside the tree trunk or dead logs of trees such as oak. During the harvesting, several trees are non-judiciously either cut down or damaged. These hamper the survivability of other non-target organisms. Regulation of such an insensible act is a must to maintain the sustainability of the resources. Moreover, there is no well-defined regulatory framework for edible insects and its use in India. This has prevented the entrepreneurs to scale up their production to an industrial scale and expanding the business in global market. Any research development related to entomophagy depends on the traditional knowledge’s of how particular insects are consumed along with its major purpose. India has no limited resources with regard to indigenous traditional knowledge; however, the lack of interest and investment from the government in research is the major hurdle to developing mass production technologies.

**Fig. 7:**
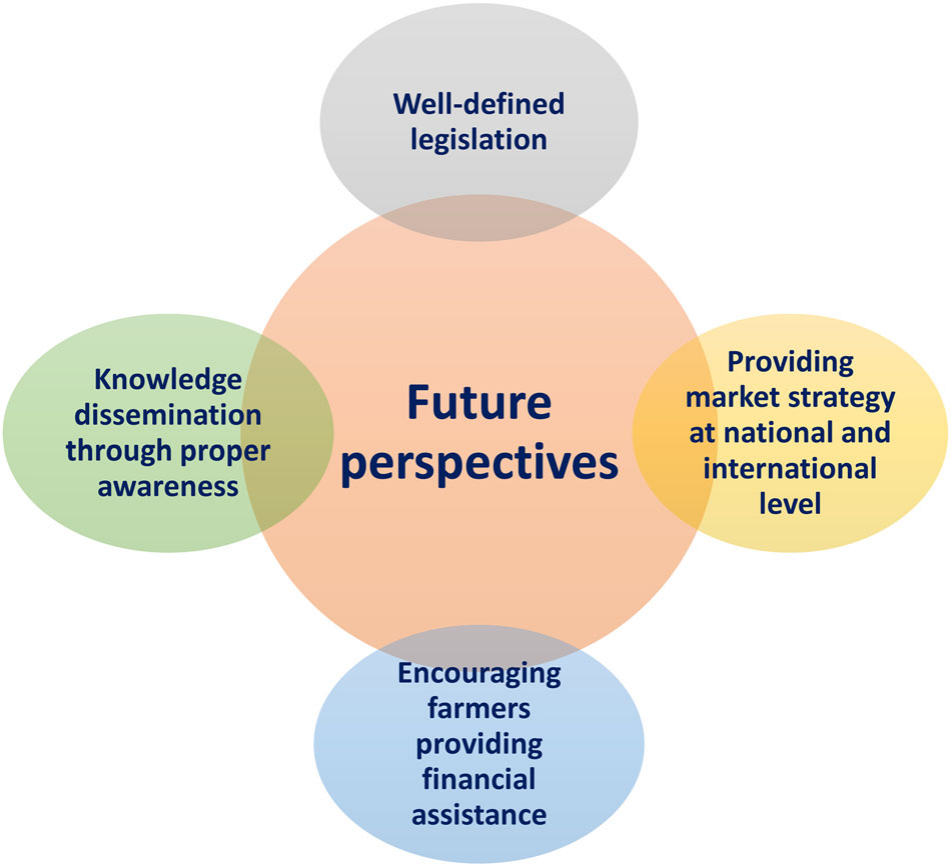
The future perspective of entomophagy in India.

Edible insect market can be a huge boon to the Indian economy because of its great diversity of edible insects. Knowledge dissemination through awareness programs, education regarding the importance of edible insects in boosting the economy and maintaining the tradition of a certain section of the population is quite essential (Fig. 7). The scientific community must play an important role in transferring technologies from lab to farm, ensuring farmers have adequate knowledge regarding the insect’s life cycle and rearing techniques. The government should encourage farmers to take up the concept of farming insects. This will reduce the overexploitation of natural resources and help in maintaining bio sustainability. Most importantly government should act fast to frame a well-defined legislation to regulate the insect market and the products that use insects as an ingredient. The government should take the initiative to set up a market strategy for small scale and medium scale insect farms. This will encourage farmers to step into this new concept of farming and also expand the existing farms.

Entomophagy tradition has been known and customary throughout the globe and diverse region wise. Countries namely Korea, Malaysia, Japan, and Tibet do not have much practice of such traditional entomophagy like in China, India, Africa, Mexico, and European countries. Species like *Apis* spp., silkworm larvae, termites, ants, bugs, few more are the most common insects practiced in entomophagy. Among thousands of entomophagy species, some species have been traditionally reported to have medicinal properties and are conserved by traditional healers of different regions of the world. Further insect farming could represent a promising way to supply alternative food sources to the increasing Indian population with high demands for edible insects. The two major hurdles that should be addressed are the non-standardized legal regulations of edible insects across the world and unpopularity of insect-based foods. Besides, from an economic point of view, it is not easy to predict the future market values and prospect of edible insects. In the future, edible insects seems to be a promising market but it requires acceptance by the public, funding for large scale industry setup, new processes and ideas for proper management of insect farming, processing, regulation and market on a trade scale.

### Reporting summary

Further information on research design is available in the Nature Research Reporting Summary linked to this article.

### Supplementary information

Reporting Summary
